# Trends in mortality and loss to follow-up in HIV care at the Nkongsamba Regional hospital, Cameroon

**DOI:** 10.1186/1756-0500-6-512

**Published:** 2013-12-05

**Authors:** Cavin Epie Bekolo, Jayne Webster, Moses Batenganya, Gerald Etapelong Sume, Basile Kollo

**Affiliations:** 1 Centre Medical d’Arrondissement de Bare, PO Box 628, Nkongsamba, Cameroon; 2 London School of Hygiene and Tropical Medicine, Department of Disease Control, Keppel Street, WC1E 7HT, London, UK; 3 Department of Global Health, University of Washington, Washington, USA; 4 World Health Organisation, Yaounde, Cameroon; 5 Department of Public Health, University of Douala, PO Box 2701, Douala, Cameroon

**Keywords:** Loss to follow-up, Mortality, HIV care, Cameroon

## Abstract

**Background:**

Access to Human Immunodeficiency Virus (HIV) care has been rolled out in Cameroon in the last decade through decentralised delivery of care and timely initiation of free antiretroviral drugs. We sought to describe the evolution of mortality and loss to follow up (LTFU) and their patient-related determinants at an HIV clinic which is facing significant challenges.

**Methods:**

A retrospective review of point of care data from HIV patients was conducted in June 2012 at Nkongsamba Regional Hospital in Cameroon to establish mortality and LTFU rates. Univariable and multivariable Cox regression models were used to screen for factors associated with the outcomes. Telephone calls were made to trace patients LTFU.

**Results:**

Between June 2005 and December 2010, 2388 HIV infected patients were admitted. Of these, 1858 were aged 15 and above and were included in our analysis. Their median age was 36 years (IQR: 30–44) and they were followed up over a total risk period of 3647.3 person-years (pyrs). The overall mortality rate was 34.6 deaths per 1000 pyrs (95% CI: 29.0-41.1) while the overall LTFU rate was 94.6 per 1000 pyrs (95%CI: 85.1-105.1).The mortality rates steadily rose to a peak of 69.6 deaths per 1000 pyrs in 2009 and then fell drastically to 20.6 per 1000 pyrs in 2010. The LTFU rate increased sharply from 29.7 in 2006 to 138.2 in 2007 and remained virtually stable until 2010. The factors associated with mortality were: being male (aHR = 2.25, 95% CI: 1.58-3.19), clinical disease progression (aHR = 2.0, 95% CI: 1.58-2.53), CD4 count <200 cells/μl (aHR = 3.14, 95% CI: 1.27-7.73), haemoglobin level <10 g/dl (aHR = 2.50, 95% CI: 1.69-3.69). Major factors associated with high LTFU rate were: distance to clinic of over 5 km (aHR = 1.25, 95% CI: 1.00-1.55), being single, having partners with unknown HIV status or taking no treatment and with CD4 count >500 cells/μl. Two- thirds (66.7%) of traced LTFU patients were dead.

**Conclusion:**

Mortality and LTFU rates in our cohort were high but there is evidence that patients’ outcomes are improving. Interventions to address factors associated with high mortality and LTFU should be implemented for optimal results in patient care.

## Background

At the end of 2012, an estimated 35.3 million people were living with HIV worldwide [1]. Sub-Saharan Africa, a region with only 12% of the global population remains the region most heavily affected by HIV accounting for about 70% of the global burden of HIV. A total of 5.2 million deaths have been averted in low- and middle-income countries between 1996 and 2012 due to increased access to antiretroviral therapy (ART) [1]. Despite this, however, mortality from HIV in Sub-Saharan Africa remains very high especially in the first year; between 8 and 26% of patients die in the first year of antiretroviral treatment, with most deaths occurring in the first few months [2]. ART has transformed HIV/AIDS into a chronic illness and a lifelong follow up for both patients in care and on ART is mandatory for optimal outcomes. Loss of patients to follow-up and care (LTFU) is, however, an important problem in resource-limited settings. A systematic review of published retention rates from ART clinics in sub-Saharan Africa (SSA) showed that the proportion of patients retained two years after starting therapy was approximately 60% [3]. Similarly, in a collaborative analysis of patients starting ART in 15 treatment programmes in Africa, Asia, and South America it was found that 21% of patients were lost to follow-up 6 months after starting ART [4]. A systematic review and meta-analysis of studies tracing patients lost to follow-up found that these patients experience high mortality compared to patients remaining in care [5]. The successful treatment of individual patients and the monitoring and evaluation of ART programmes both depend on regular and complete patient follow-up.

Cameroon with an estimated population of 19.4 million in 2010 [6] had an HIV prevalence rate in 2011 of 4.3% down from 5.5% in 2004 [7]. The ART coverage increased from 36.5% in 2010 to 49.6% in 2011 [8]. The country HIV Drug Resistance Working Group reported that the proportion of patients lost to follow up varied between 7 to 77% across various treatment sites in 2010 with a national average of 33%, higher than the reported rate (25%) in 2008 [9].A high level of drug resistance was reported in a cohort of naïve and ART treated patients infected with HIV-1 only after few years of treatment in Yaounde, Cameroon probably because of high loss to follow up and poor compliance [10]. In 2005 the Douala Antiretroviral Initiative (DARVIR) had identified cost of ARV as the main risk factor for LTFU [11]. Late presentation and or late ART initiation [12], TB co-infection [13] and long travels to clinic [14] were the other important factors for treatment interruption reported by different authors. These studies were mostly conducted before 2007 during an era of very constrained access to ARV, and in urban settings. Since 2007 several changes have been instituted: ARVs are dispensed free of charge to patients, many treatment centres have been established nationwide, treatment has been decentralised, ART initiation cut off has been increased from 200 to 350 and the more toxic stavudine (D4T)-based regimen has been gradually phased out since 2010 in accordance with the 2010 World Health Organisation (WHO) recommendations [15]. These changes were instituted to improve access to HIV care. Consequently, an increased number of patients are receiving ART [16,17] but the outcomes of these patients before and after these interventions are not known in terms of morbidity and mortality.

Mortality and Loss to follow up in HIV programs are on-going challenges to the scaling up of HIV management. The rates and determinants of which vary by health facility [18] and as such each setting needs to determine theirs and institute improvement programs.

This study sought to use routine clinic data to document trends in Mortality and Loss to Follow-up in HIV Care at Nkongsamba regional hospital where the highest proportion of LTFU (77%) had been documented in an earlier study [9].

## Methods

### Study site

The study was conducted at the Regional Hospital of Nkongsamba, Moungo Division of the Littoral Region of Cameroon. It is a 2nd level reference public health facility with a catchment area of over 321,295 inhabitants. The HIV clinic was established in 2005 and offers voluntary HIV counselling and testing (VCT), ART and limited community outreach services to patients on ART.

### Ethical aspects

The study was approved by the London School of Hygiene and Tropical Medicine (LSHTM) Ethics Committee. Permission to conduct the study was provided by The Littoral Regional Delegate for Public Health in Cameroon and The Director of The Nkongsamba Regional Hospital. It was not possible or practical to obtain consent from each of the participants. Since patient identifying data was abstracted, this is considered a low risk.

### Definitions

Loss to follow-up: a patient was classified as LTFU if there was no contact for 90 days after the last missed appointment for ARV refill. For patients not yet on ART, 180 days of no contact after the last clinical or laboratory appointment was considered as LTFU. LTFU is an early warning indicator for HIV drug resistance. WHO considers a proportion of LTFU above 20% to be high. In this study, the LTFU rate was defined as the number of patients LTFU during the follow up period divided by the total person-years at risk.

Mortality: all cause death of an HIV patient enrolled in care. The mortality rate was defined as the number of deaths occurring during the follow-up period divided by the person-years at risk taking into account LTFU and transfer outs in the denominator.

### Inclusion criteria

Adults of both sexes aged 15 years old and above, tested positive for HIV-1, recruited into the clinic cohort between 2005 and 2010.

### Sample size

A sample size of 680 was needed to detect a 5% difference in LTFU between the sub-categories of a variable based upon the national average in 2010 of 33% LTFU.

### Study design

We conducted an exploratory retrospective cohort study of HIV positive patients recruited into the clinic from 20th June 2005 to 31st December 2010 and followed up till 14th June 2012. In this cohort, the rate of occurrence of events (deaths and LTFU) was measured over time and the putative determinants of these events were compared between the group which experienced these events and the group that did not. Death was ascertained by relatives who informed the health workers, active tracing by community health workers or by telephone calls.

### Data collection

We used data from the HIV clinic pre-ART and ART registers and individual patient medical records. The registers were designed by the National AIDS Control Committee (NACC) for the standardised collection and reporting of data. From pre-ART and ART registers, we counted the number of patients ever enrolled in care, the number initiated on ART and cotrimoxazole prophylaxis, their dates of ART initiation, the type of regimen, drug refill dates and the patient status. The medical records were designed by the hospital and are stored in the clinic’s records office by year of recruitment, by codes and by patient outcome.

From each of the patient’s medical records we abstracted the following information: socio-demographic characteristics which were telephone contact numbers, date of birth, gender, place of residence, occupation, insurance scheme, religion, distance to clinic, alcohol and tobacco consumption, education and matrimonial status. Clinical features including date of HIV diagnosis, baseline weight and height, disease and clinical stage at presentation were also collected. Laboratory parameters at baseline and during follow-up such as CD4 count, haemoglobin level, liver transaminases, fasting blood sugar, creatinine and urine albumin levels were also used. Treatment related variables including date of ART initiation, first line ART regimen, drug side effects, cotrimoxazole prophylaxis and alternative therapies were noted. Outcome measures of interest included date of exit from the cohort, the nature of the event marking the exit be it death, transfer out, loss to follow up or continuing care till 14th June 2012, the day of censorship.

Treatment registers were used to ascertain the last day a patient was seen in the clinic. We dialled the available phone numbers and where reachable, we proceeded to find out if they were alive, dead and date of death, if alive and taking treatment elsewhere or not, reasons for defaulting and then advised them to return to care were appropriate. These variables were entered electronically into a questionnaire created using the Epidata® software version 3.1. The final dataset with variables of interest was exported to Stata® software for analysis.

### Data analysis

Data analyses were performed using Stata® 12.1(StataCorp LP, TX77845, USA). The data set was checked for logical inconsistencies, illegal codes, omissions and improbabilities by tabulating, summarising, describing and plotting variables. Missing observations were excluded where they constituted a small random proportion but were included if they were found to be differential amongst subgroups.

Our outcomes of interest were deaths and LTFU analysed as time-to-event variables. Putative risk factors of interest for Mortality and LTFU, how they were treated or recoded and the rationale for categorisation are presented in Table 1. Age, year of enrolment and gender were considered *a priori* confounders. No effect modification between variables was considered.

**Table 1:** Categorisation of explanatory variables

**Factor**	**Type of variable and categorisation**	**Rationale for categorisation**
Calendar year of entry	Ordered categorical:	Events, program performances usually vary with time
	2005, 2006, 2007, 2008, 2009, 2010	
Current age groups (years)	Ordered categorical: 15 – 24, 25 – 34, 35 – 44 , >45 (using Lexis expansion)	Events vary with age and adjusting for age attained in cohort is more appropriate than by age at entry as this avoids residual confounding
Gender	Binary: Females, Males	Gender is usually associated with most diseases and thus a strong confounder
Region or province	Unordered categorical: Littoral, South-West, West, Other	Risk of clustering of events by place of residence
Marital status	Unordered categorical: Single, Monogamous, polygamous, divorced, widowed	Socio-cultural and economic empowerment is usually differential in African context
Partner HIV Status	Unordered categorical: Negative	Health seeking behaviour, adherence, treatment success or failure may be determined by partner HIV status, viral load and viral strain
	Positive but not taking ART, Positive and taking ART, Unknown	
Occupation	Binary: Lower grade, higher grade	Proxy measure of level of socio-economic status. Grading based on International Standard Classification of Occupations (ISCO)
Distance(km)	Binary: ≤5, >5	Usual walking distance within 30 minutes to health facility is 5 km (indicator of accessibility); also reflects the population living in the urban centre and usually accessible to community workers
Alcohol Intake	Binary: No, Yes	Interaction with drugs, co-morbidity, behaviour change
Smoking	Binary: No, Yes	Factor of many co-morbid conditions
WHO Clinical Stage	Ordered categorical:	Risk of death and health care seeking behaviour is influenced by the severity of disease usually; the staging also guides when to start ART
	I = Asymptomatic condition	
	II = Mild	
	III = Advanced	
	IV = Severe	
Immune deficiency	Ordered categorical: based on CD4 count	Risk of opportunistic infection and thus death depend on CD4 count; initiation of ART also depends on CD4 count
	None: ≥500	
	Mild: 350-499	
	Advanced: 200-350	
	Severe: <200	
Haemoglobin level (g/dl)	Binary: <10, ≥10	Cut off point based on the median value in this HIV cohort to define anaemia
Alanine amino-Transferase, ALAT (IU/l)	Binary: <50 , ≥50	Cut off point as determined by the hospital laboratory
		Above which indicates liver injury (more specific marker)
Aspartate amino-Transferase, ASAT (IU/l)	Binary: <45, ≥45	Cut off point as determined the hospital laboratory
		Above which indicates liver injury(less specific marker)
Fasting blood sugar (mg/dl)	Binary: <126, ≥126	Cut off point above which defines diabetes mellitus
Creatinine level(mg/l)	Binary: ≤15, >15	Cut off point as determined the hospital laboratory
		Above which indicates kidney injury
NNRTI Regimen	Binary: EFV(efavirenz)-based, NVP(nevirapine)-based	EFV or NVP is present in all first line regimens; their relative efficacy, tolerance or toxicity may be relevant
NRTI Regimen	Unordered categorical: ABC(abacavir)/TDF(tenofovir)-based, AZT(zidovudine)-based,	NRTI form the backbone of all first line regimens; while 3TC is invariably present, the relative toxicity, efficacy and tolerance may be relevant to adherence and emergence of drug resistance and thus to Mortality and LTFU
	D4T(stavudine)-based, None (missing value)	
Drug Change	Binary: No, Yes	Proxy measure for the presence of drug toxicity (usually) or drug resistance (rarely)
Cotrimoxazole Prophylaxis	Binary: No, Yes	Measure to prevent common opportunistic infections

NNRTI: non-nucleoside reverse transcriptase inhibitor, NRTI: nucleoside reverse transcriptase inhibitor.

Summary statistics were presented as proportions for categorical variables and as means (standard deviations) or medians (IQR-Interquartile Range) for continuous variables. Pearson Chi-square analyses were used to examine the difference in proportion of deaths and LTFU between the various subgroups created in Table 1. A t-test was used for the difference between means in two subgroups. Kaplan-Meier survival curves were used to display the risk of mortality and LTFU over the study period. Classical cohort analysis was used to obtain Mortality and LTFU rates and their 95% confidence intervals (95%CI). These rates were plotted to display the trend over the years. A univariable Cox regression model was set up to screen for factors associated with Mortality and LTFU. Crude hazard ratios (HR) and their 95%CI were obtained. The p-values for hypotheses testing were calculated from likelihood ratio tests (LRT). A test for linearity and departure from linearity was performed for ordered categorical variables. Variables found to be associated at 5% confidence level, with Mortality and LTFU were included in a multivariable Cox model to adjust for the effects of gender, current age and calendar year. The proportionality hazard assumption over time was assessed graphically using Aalen plots. Where the model could not converge due to collinearity, the variables responsible were taken out of the model.

From the established list of LTFU patients, we counted the number that could be traced, their ultimate outcomes and reasons for dropping out. The results were presented as proportions. Similarly, the number of patients ever enrolled and those who ever started ART was presented graphically as absolute numbers.

## Results

### Participants

Results for 1858 adults are presented. Of these 1305(70.2%) were females. Most patients 483 (26.5%) were recruited in the year 2008. The median age at presentation was 36 years (IQR: 16–70). A total of 3647.3 person-years of observation are reported. The majority of them (92.3%) were residing in the Littoral Region but just 43.5% had to travel 5 km or less to the clinic (Table 2). About 52% of them were peasant farmers and only 7% were currently involved in a high grade job. Sixty two per cent (1095) of patients were in WHO Clinical Stage III and IV, they had a median baseline CD4 count of 201 cells/μl (IQR: 92–304) and a baseline median haemoglobin level of 10.9 g/dl (IQR: 9.2-12.5).

**Table 2:** Baseline characteristics of patients

**Factor**	**Number**	**Factor**	**Number**
	**N (%)**		**N (%)**
**Calendar year**			
2005	103(5.7)	**Immune deficiency**	
2006	208(11.4)	None	234(13.4)
2007	401(22.0)	Mild	186(10.7)
2008	483(26.5)	Moderate	449(25.8)
2009	351(19.2)	Severe	871(50.1)
2010	278(15.2)	Total	1740(100.0)
Total	1824(100.0)		
**Current age groups (years)**			
15 – 24		**Haemoglobin level(g/dl)**	
25 – 34		> = 10	848(61.1)
35 – 44	671(36.3)	<10	539(38.9)
45+	579(31.3)	Total	1387(100.0)
Total	450(24.3)		
	1824(100.0)		
**Gender**		**Alanine amino-**	
Females	1284(70.4)	**Transferase(IU/l)**	
Males	540(29.6)	<50	1344(98.3)
Total	1824(100.0)	> = 50	23(1.7)
		Total	1367(100.0)
**Region**		**Aspartine amino-**	
Littoral	1661(92.3)	**Transferase(IU/l)**	
South-West	44(2.4)	<45	1311(95.9)
West	79(4.4)	> = 45	56(4.1)
Other	16(0.9)	Total	1367(100.0)
Total	1800(100.0)		
**Marital status**			
Single	568(31.8)	**Fasting blood sugar(mg/dl)**	
Monogamous	636(35.6)	<126	996(94.1)
Polygamous	152(8.5)	> = 126	62(5.9)
Divorced	141(7.9)	Total	1058(100.0)
Widowed	289(16.2)		
Total	1786(100.0)		
**Partner HIV**			
Negative	217(12.1)	**Creatinine level(mg/l)**	
Positive-ART	220(12.2)	<=15	125(82.8)
Positive + ART	99(5.5)	>15	26(17.2)
Unknown	1262(70.2)	Total	151(100.0)
Total	1798(100.0)		
**Occupation**		**NNRTI Regimen**	
Lower grade	1668(93.0)	EFV-based	395(33.7)
Higher grade	126(7.0)	NVP-based	779(66.3)
Total	1794(100)	Total	1174(100.0)
		**NRTI Regimen**	
**Distance(km)**			
≤5	781(43.5)	ABC/TDF-based	19(1.0)
>5	1015(56.5)	AZT-based	527(28.9)
Total	1796(100.0)	D4T-based	628(34.4)
		None	650(35.6)
		Total	1824(100.0)
**Alcohol**		**Drug change**	
No	687(39.0)	No	804(68.3)
Yes	1077(61.0)	Yes	374(31.7)
Total	1764(100.0)	Total	1178(100.0)
**Smoking**		**Cotrimoxazole use**	
No	1495(84.7)	No	72(5.4)
Yes	269(15.3)	Yes	1251(94.6)
Total	1764(100.0)	Total	1323(100.0)
**WHO Clinical stage**			
I	404(22.9)		
II	266(15.1)		
III	955(54.1)		
IV	140(7.9)		
Total	1765(100.0)		

The three commonest modes of presentation were, constitutional syndrome [unexplained wasting, fever and diarrhoea for over 1 month] (29%), chest infections including tuberculosis (20%) and referral from antenatal clinics (13%). At least 95% of patients had normal baseline liver transaminases while 82.8% of patients had a normal creatinine level of <15 mg/l. The proportion of patients who had not yet started ART was 35.6% meanwhile among those on ART, about two out of three were on nevirapine-based regimens and just over a half (53.5%) were taking stavudine-based regimens. Only 1% was placed on either abacavir- or tenofovir –based therapies. Over two-thirds of patients on treatment did not experience a drug substitution for any reason. Almost 95% of the whole cohort was receiving cotrimoxazole prophylaxis.

### Outcome assessment

**Figure 1:**
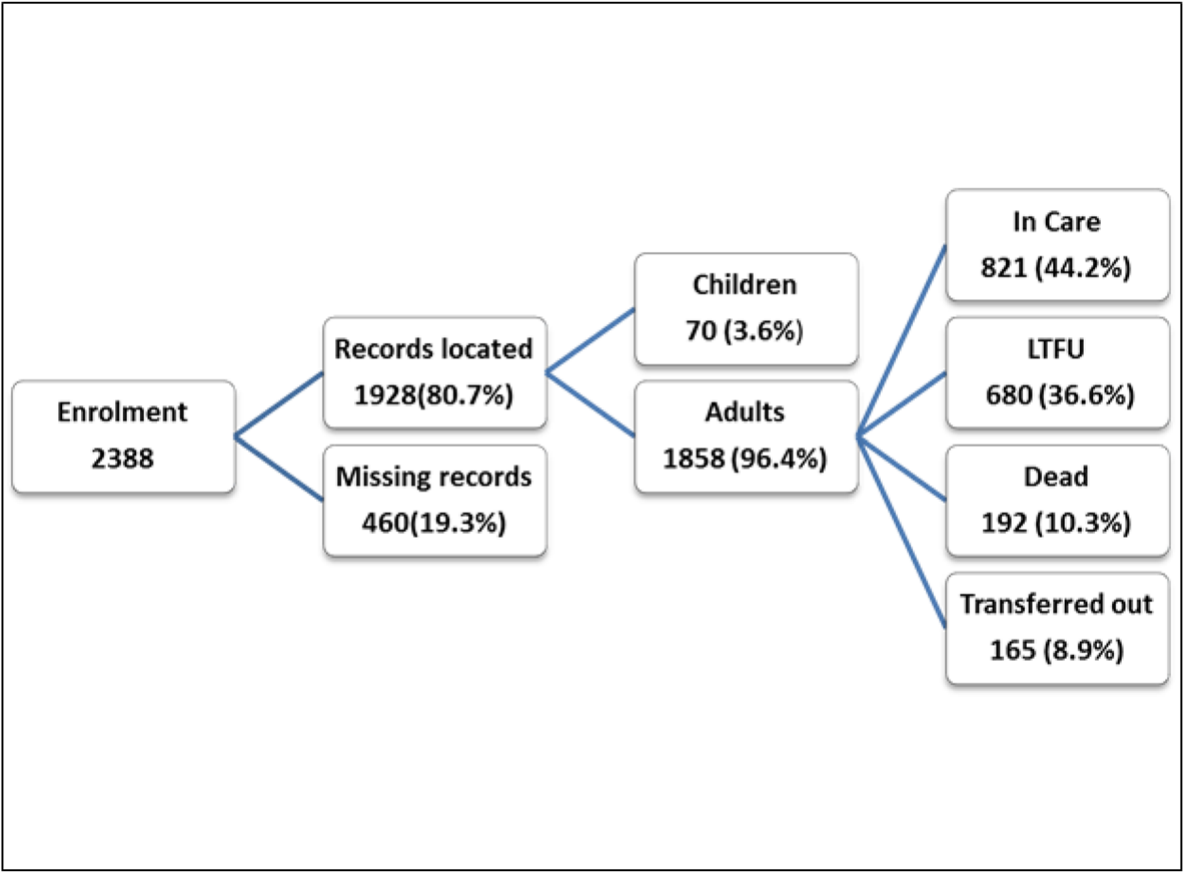
Cohort construction of participants enrolled between 2005 and 2010.

Over the seven-year follow up period, amongst the 1858 enrolled, 192(10.3%) were confirmed dead, 680(36.6%) had been LTFU, 165(8.9%) were confirmed to have been transferred out meanwhile 821(44.2%) were still continuing care by June 2012 (Figure 1).

**Figure 2:**
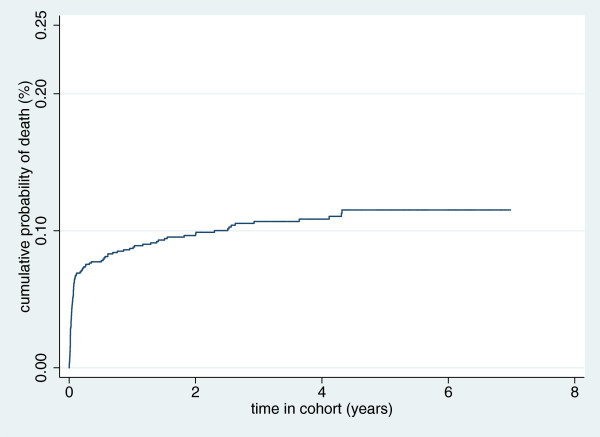
Kaplan-Meier curve of cumulative probability of death.

**Figure 3:**
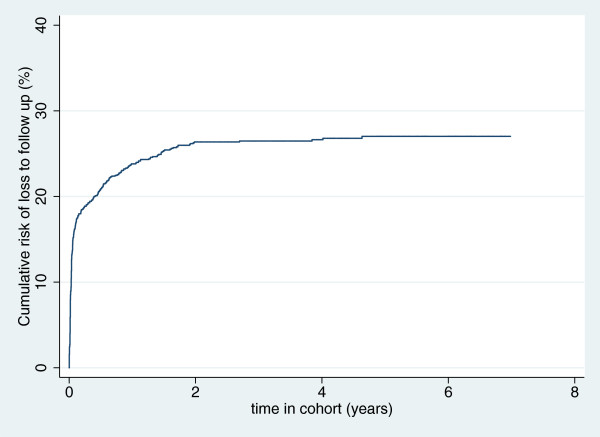
Kaplan-Meier curve of cumulative probability of loss to follow-up.

The survival probabilities over time and the risks of LTFU over time as displayed by Kaplan-Meier curves (Figures 2 and 3 respectively), show that the risk of LTFU in the first 12 months in care is above 20% while the risk of death within two years of enrolment is about 10%.

The overall mortality rate was 34.6 deaths per 1000 person-years (95%CI: 29.0-41.1). This rate initially dropped between 2005 and 2006 then increased gradually from 2006 to 2009 (from 24.6 to 69.6) before decreasing in 2010 to 20.6 per 1000 pyrs.

**Figure 4:**
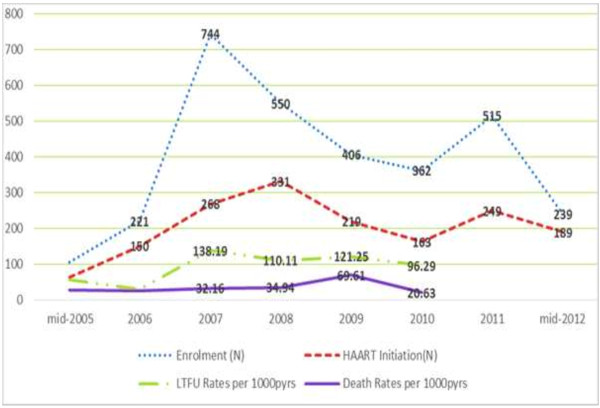
Trends in enrolment into care, Initiation on ART, LTFU and Mortality.

The overall LTFU rate was 94.6 per 1000 person-years (95%CI: 85.12-105.12). It increased sharply from 29.7 in 2006 to 138.2 in 2007 and remained virtually stable until 2010. Both Mortality and LTFU rates broadly reflect the trend in enrolment and ART initiation during the same time frame (Figure 4).

### Predictors of mortality

In univariable analysis, the mortality rate was just over two times higher in men than in women HR = 2.25(95%CI: 1.58-3.19, p < 0.0001) and the age group 25–45 years was most affected by death. Patients living more than 5 km from the clinic had a 38% reduction in mortality compared to those living within 5 km to the clinic HR = 0.62(95%CI:0.43-0.88, p < 0.007). Patients with late clinical presentation (stages III and IV) had the poorest outcomes. The rate of death doubled from one clinical stage to another (HR = 2.0, 95%CI: 1.58-2.53; LRT for linearity, X^2^ on 2df, p = 0.30) showing a dose–response effect. The mortality rate was highest in those with severe immune deficiency, those with anaemia (HR = 1.99, 95%CI: 1.37-2.87) and those who smoked (HR = 2.06, 95%CI: 1.37-3.08). However, patients treated with a nevirapine-based regimen (HR = 0.37, 95%CI: 0.25-0.56, p < 0.0001) and those whose treatment was ever changed (HR < 0.01, p < 0.0001) had better outcomes. There was no association between mortality and either marital status, partner HIV status, socio-economic status, alcohol intake, liver and kidney function abnormalities, type of NRTI drug used or cotrimoxazole preventive therapy (CPT) use. In multivariable analysis (Table 3), distance to clinic (aHR = 0.60, 95%CI: 0.42-0.86), clinical stage and immunological status, low haemoglobin (aHR = 2.50, 95%CI: 1.69-3.69), and choice of NNRTI drug remained significantly associated with mortality after adjusting for gender, current age and time.

**Table 3:** Factors independently associated with mortality

**Factor**	**Crude hazard ratios (95% Confidence intervals)**	**P-values from LRT**	**Adjusted hazard ratios (95% Confidence interval)**	**P-values from LRT**
**Distance (km)**				
≤5	1.00	0.007	1.00	
>5	0.62 (0.43-0.88)		0.60 (0.42-0.86)	0.005
**Gender**				
Females	1.00	<0.0001	1.00	
Males	2.25 (1.35-3.19)		2.25 (1.35-3.19)	<0.0001
**Smoking**				
No	1.00	0.001	1.00	
Yes	2.06 (1.37-3.08)		1.44 (0.92-2.27)	0.11
**WHO Clinical**				
**Stage**				
I	1.00		1.00	
II	1.18 (0.48-2.91)	<0.0001	1.18 (0.47-2.93)	<0.0001
III	3.77 (1.95-7.26)		3.55 (1.82-6.91)	
IV	6.38 (2.99-13.63)		6.13 (2.82-13.29)	
**Immune deficiency**				
None	1.00	<0.0001	1.00	
Mild	1.07 (0.31-3.70)		1.13 (0.33-3.91)	
Moderate	0.88 (0.32-2.43)		0.81 (0.29-2.25)	<0.0001
Severe	3.62 (1.48-8.90)		3.14 (1.27-7.73)	
**Haemoglobin**				
**level(g/dl)**				
≥10	1.00	0.0003	1.00	
<10	1.99 (1.37-2.87)		2.50 (1.69-3.69)	<0.0001
**NNRTI Regimen**			1.00	
EFV-based	1.00	<0.0001	0.40 (0.26-0.61)	<0.0001
NVP-based	0.37 (0.25-0.56)			

### Predictors of loss to follow up

In univariable analysis, we found an association between LTFU and year of recruitment, place of residence, marital and partner HIV status, CD4 count, and ART regimen and cotrimoxazole prophylaxis. However, after adjusting for time spent in cohort (Table 4), place of residence, marital status and partner’s HIV status were associated with LTFU but with no clear pattern or direction of effect. Living more than 5 km away from the clinic (aHR = 1.25, p = 0.01) was associated with a higher risk of LTFU. Receiving ART was linked to a 25 – 81% chance of LTFU depending on the ARV regimen. Meanwhile, receiving cotrimoxazole prophylaxis (aHR = 0.47, p < 0.0001), having had a drug substitution/switch (aHR = 0.07, p < 0.0001) and receiving a nevirapine-based regimen (aHR = 0.75, p = 0.04) were associated with a reduction in the rate of LTFU.

**Table 4:** Adjusted risk ratios for factors independently associated with LTFU

**Factor**	**Unadjusted Cox hazard ratios**	**P-values from LRT**	**Adjusted hazard ratios**	**P-values from LRT**
	**(95% Confidence intervals)**		**(95% Confidence intervals)**	
**Calendar year**				
2005	1.00		1.00	
2006	0.53(0.29-0.97)		0.48(0.26-0.87)	
2007	1.69(1.05-2.72)		0.94(0.57-1.55)	<0.0001
2008	1.21(0.75-1.96)	<0.0001	0.89(0.54-1.47)	
2009	0.99(0.56-1.66)		0.96(0.58-1.59)	
2010	0.60(0.34-1.06)		0.45(0.25-0.78)	
**Age group (years)**				
15 – 24	1.00		1.00	
25 – 34	0.62(0.43-0.89)	0.06	0.67(0.46-0.97)	
35 – 44	0.67(0.47-0.97)		0.71(0.49-1.04)	0.21
45+	0.60(0.41-0.88)		0.66(0.44-0.98)	
**Region**				
Littoral	1.00		1.00	
South-West	1.29(0.67-2.50)	0.02	1.32(0.33-5.32)	
West	0.36(0.16-0.81)		1.69(0.36-7.83)	0.03
Other	0.77(0.19-3.11)		0.49(0.10-2.45)	
Marital status**				
Single	1.00		1.00	
Monogamous	0.65(0.50-0.84)		0.66(0.51-0.85)	
Polygamous	0.60(0.39-0.93)	0.005	0.59(0.38-0.92)	0.006
Divorced	0.93(0.63-1.35)		0.92(0.63-1.35)	
Widowed	0.67(0.49-0.93)		0.67(0.49-0.93)	
Partner HIV **				
Negative	1.00		1.00	
Positive-ART	0.62(0.37-1.02)		0.61(0.37-1.01)	
Positive + ART	0.77(0.42-1.40)	0.0003	0.72(0.40-1.30)	0.0005
Unknown	1.29(0.92-1.81)		1.24(0.89-1.74)	
Distance(km)*				
≤5	1.00		1.00	
>5	1.22(0.98-1.51)	0.07	1.25(1.00-1.55)	0.01
**NNRTI Regimen**				
EFV-based	1.00		1.00	0.04
NVP-based	0.77(0.59-1.01)	0.06	0.75(0.57-0.99)	
**NRTI Regimen**				
ABC/TDF-based	0.13(0.02-0.93)		0.19(0.03-1.39)	
AZT-based	0.64(0.49-0.83)	<0.0001	0.59(0.45-0.77)	
D4T-based	0.55(0.43-0.71)		0.55(0.42-0.71)	<0.0001
None	1.00		1.00	
**Immune deficiency**				
None	1.00		1.00	
Mild	0.76(0.51-1.14)	<0.0001	0.75(0.50-1.13)	<0.0001
Advanced	0.43(0.30-0.60)		0.42(0.30-0.59)	
Severe	0.55(0.41-0.75)		0.54(0.40-0.74)	
**Drug change**				
No	1.00		1.00	
Yes	0.07(0.04-0.12)	<0.0001	0.07(0.04-0.12)	<0.0001
**Cotrimoxazole**				
**Prophylaxis**				
No	1.00	0.004	1.00	<0.0001
Yes	0.45(0.30-0.75)		0.47(0.30-0.74)	

*adjusted for time and place of residence **adjusted for each other and time.

### Outcome of patients lost to follow up

**Figure 5:**
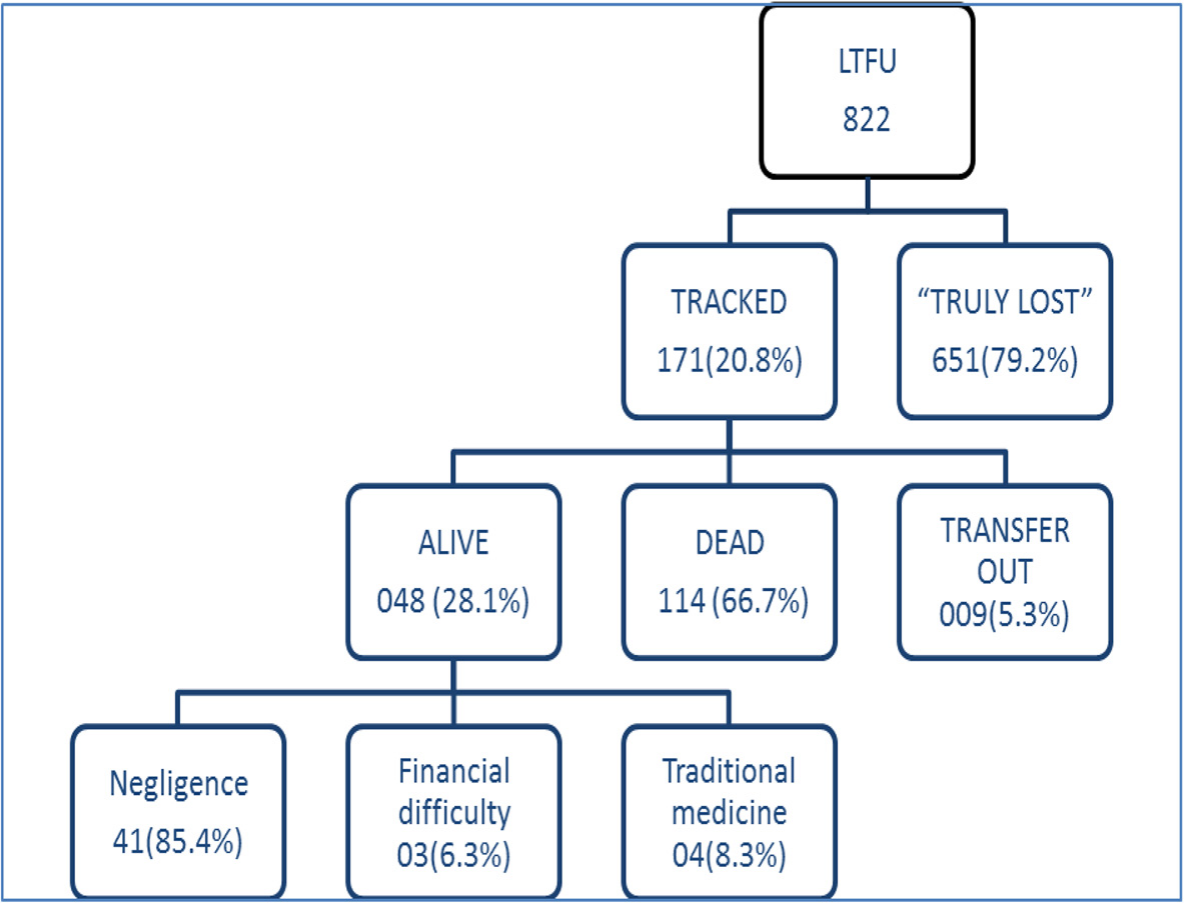
Outcome of patients lost to follow-up.

Amongst the persons LTFU, only 20.8% could be traced of which two-thirds were confirmed by relatives to have died. The majority (85.4%) of those still alive ( including those who were receiving and not receiving ART) were not motivated to turn up for their appointments but all of them did promise to return for follow up (Figure 5).

## Discussion

This study has demonstrated high rates of mortality and LTFU in the early years of the program but has also shown a trend towards reduction over the years. Mortality of HIV-infected patient was 28.3 per 1000 pyrs in 2005, increased to 69.6 per 1000 pyrs in 2009 and then sharply decreased to 20.6 per 1000 pyrs in 2010.This trend was also documented for LTFU. The rate at the start of the program in 2005 was 56.6 per 1000 pyrs, rose to 138.2 per 1000 pyrs in 2007 before gradually showing a gradual reduction to a rate of 96.3 per 1000 pyrs in 2010.

### Trend in mortality, LTFU, enrolment and ART initiation

The number of people under HIV care and ART increased tremendously in the year 2007 which marked the beginning of free ARV, reduction in the cost of baseline laboratory tests and at a time when the Nkongsamba Regional Hospital was the only HIV clinic in the municipality and its environs. The steady fall in the number of people enrolled from 2008–2010 may be due to the further expansion of the ART program to nearby district hospitals and a fall in the prevalence of HIV in the country [7]. The second peak in 2011 may be the result of the revision of National and WHO guidelines in 2010 whose main highlight was early ART initiation [15]. This period was also marked by a renewed and substantial support to the country program by the Global Fund. The enrolment data in 2005 and 2012 are truncated.

This increase in access to care is universal and is associated with a decrease in the number AIDS-related deaths but with variations from one region to another [19]. In this study, the Mortality and LTFU rates between 2005 and 2010 mimic the trend in enrolment overall, but with some variation. The poor performance can be due to the increase in the number of patients in a resource constraint setting. It is important to note that, this cohort of patients in the clinic is in the hands of three dedicated nurses and two community relay agents. The doctors, pharmacy clerks and laboratory technicians only dedicate part of their time to the HIV clinic as they equally attend to all patients seen daily in the hospital for other conditions. Between 2008 and 2009, drugs and laboratory reagents stock out were frequent and the activity of community workers suspended. This could explain why LTFU and mortality were at their peaks. In 2009, the program was hit by a financial crisis after the country failed to qualify for funding from the Global Fund. It is not clear if the peak in Mortality and LTFU rates in 2009 could be linked to the crisis as data from other clinics in the country are not available for comparison. The improvement observed in 2010 may just reflect a fall in enrolment or a real impact from renewed national and multilateral effort. These system-related factors are not examined in this study but as actors in the health care system, it is our opinion and can be a plausible explanation of the trend observed. A study to examine these structural and contextual factors might be advisable just as would be extending the current study to measure the rates in 2011 and 2012 to see if the rates continue to decrease as observed from 2010.

Although the LTFU as a proportion or risk is high (36.33%) in 2010, it is far below the 77% reported by Billong *et al*. in 2010 from the same clinic [9]. Our LTFU rate for 2010 was 96.29 per 1000 person-years. A rate may be the appropriate measure of incidence since patients are enrolled at different times (staggered entry) and have different risk periods. Though the difference in approach may lead to different results, we strongly believe that, our estimate is more plausible. Our Mortality and LTFU rates although corrected for time spent in cohort, may be limited by measurement bias owing to transfer outs, incomplete ascertainment of the outcome of patients LTFU, survival estimates not corrected for LTFU [20,21] and the absence of sensitivity analysis for our regression model. The effect on our estimates will likely be an underestimation.

### Determinants of mortality

This study identified age, sex, calendar year, distance to clinic, clinical and immune status at presentation, low baseline haemoglobin concentration, choice of NNRTI (Non-Nucleotide Reverse Transcriptase) drug and drug substitution to be strongly associated with mortality.

Age is often associated with diseases and death. The age group 25–45 had the highest mortality rate probably because the disease is more common in this age bracket, the sexually active population. Men experienced more deaths than women though more women were affected by the disease. A likely explanation for this may be due to the fact that older men were significantly more affected by disease compared to women (mean age of 41.6 years for men against 35.9 years for women) and men presented at a later stage than women (68% of men against 59% women presented with advanced disease). Women present early owing to antenatal care referrals. Patients living within 5 km of the clinic had a higher mortality rate than those further away. The evidence was however weak and this could be due to the fact that this category of the population who live in the urban centre of Nkongsamba came to die in the hospital leading to increased ascertainment of their outcome compared to the distant rural population who mostly die at home (ascertainment bias). This argument is further supported in this study by the evidence that the LTFU rate was higher in those from rural areas. If our results are valid, then the likely explanation for the observation that those living closer to the hospital suffered more deaths could be that they may be less adherent to treatment due to fear of stigma from users and health workers familiar to them. A more complete investigation into the outcome of patients LTFU might help reduce this ascertainment bias. It is well known that the natural history of HIV/AIDS follows the clinical and immunological stages with more deaths occurring at later stages. Late presentation was common in this cohort and as suggested by the findings of this study is a driving force for mortality. A low haemoglobin concentration (anaemia) was another major factor contributing to more deaths. Low haemoglobin is related to chronic inflammation owing to HIV *per se* but is further compounded by poor nutrition and concurrent parasitic infestations including intestinal worms and malaria that are common in Africa [22]. Correcting severe anaemia (haemoglobin < 8 g/dl) is indispensable before the start of ART. This correction is done in clinical practice by blood transfusion and or prolonged nutritional support and supplementation with haematinics. The time and money necessary for treating anaemia adds to the misery of patients already heavily afflicted. Smoking though strongly associated with mortality in univariable analysis, its effect was confounded by gender as smokers were mainly men (76%). Many smokers also may have stopped smoking at the time of this study as a result of illness. Patients taking Nevirapine (NVP) in their combination of drugs had better outcomes than those on Efavirenz (EFV) [HR = 0.4(0.26-0.61), p < 0.0001]. This result is contrary to an accumulation of evidence indicating that EFV has a superior efficacy and tolerability compared to NVP [23-25]. Our results might be attributed to the fact that EFV is more expensive and was usually recommended to the subgroup of patients with severe disease who had a higher risk of death. EFV was contraindicated in women who were pregnant or who planned to become pregnant. (EFV is recently recommended in pregnant women [WHO 2012 update]) [26]. Patients who had one or more drugs in their combination substituted had better outcomes. Drug substitution was done in an event of severe side effects so much so that in 2010–2011 all patients on Stavudine (D4T) were switched to either Zidovudine or Tenofovir in line with the WHO 2010 guidelines recommending the phasing out of D4T [15]. It is difficult to attribute increased survival to these changes *per se* as the close monitoring these patients (with side effects) received is likely to have impacted on their outcomes.

Other baseline laboratory tests (transaminases, blood sugar and creatinine) were not associated with survival. These indicators are not linked to HIV pathology but used to monitor or guide treatment. Given that they constitute a financial barrier to the initiation of ART and are not directly associated with AIDS-related deaths, it might be necessary to either drop them or use them in a case by case scenario. Alcohol intake is widespread and most of the patients (61%) in the cohort used alcohol. Categorising alcohol intake into units of consumption and assessing these could be a more useful indicator. Cotrimoxazole reduces the risk of bacterial infections and malaria in persons living with HIV [27] and was given free of charge to a majority of patients and this explains why it was not associated with mortality in this study. Although evidence for an association between partner HIV status, marital status and mortality was very weak, it is however plausible that patients whose partners are receiving ART do have better outcomes because of mutual assistance to improve adherence and thus reduced risk of emergence of drug resistance. Similarly, matrimonial status was not a determinant but it is plausible that those engaged in polygamous marriages had poorer outcomes probably due to cross infection and re-infection. Those living out of the littoral region in which the clinic is based also had poorer survival outcomes and this might be as a result of distance although there was no evidence in favour of region of residence as a factor.

### Determinants of loss to follow up

Factors for which there was suggestive or strong evidence for an association with LTFU after adjusting for time in cohort include calendar year (proxy for number enrolled), region of residence, distance to clinic, marital and partner HIV status, ART and cotrimoxazole prevention therapy ( CPT).

It is probable that persons living further from a health facility will have less access to health care because they have to put in an extra financial and motivational effort. Patients living closer to and at walking distance from the health facility were less likely to discontinue follow up care. Similarly, patients residing in regions out of the Littoral Region in which our clinic is situated were more likely to drop out. Our findings are consistent with an earlier study in the South West Region of Cameroon [14]. However, those living in the furthest regions appear to have better retention rates. This is unusual and the likely explanation may be that, they constitute a minority subgroup mostly workers who originate from a locality close to the clinic but because they live very far away, their appointments for drug refill are spaced out to up to three months. This preferential treatment sounds unethical, but it is a strategy used and accepted locally to reduce transport cost and job absenteeism for these far-distant patients. Patients receiving any form of treatment (ART and or CPT) from the clinic were more likely to remain in care. Patients with advanced or severe immune deficiency were more likely to remain in care probably because they themselves and even health workers were too concerned by their illness and were usually those on both ART and CPT. Similarly, patients with mild or no immune suppression may believe they are not too sick to seek regular attention about their health and thus more likely to lose contact with the health facility. This raises concerns about patient education or counselling on asymptomatic HIV disease.

Though evidence for an association between age and LTFU was weak, it is nonetheless plausible to observe that the youngest age group is most likely to drop out because they are usually asymptomatic at presentation and consequently not too much worried about their present condition. Age was likely to be confounded by disease stage.

### Outcome of patients lost to follow up

A phone call was the optimal means of reaching and tracing patients LTFU (the use of community workers was an alternative but prohibitively expensive). Given that half of the 402 patients with no phone contacts were those LTFU and that up to 66.7% of those LTFU for whom information was available were already dead, it is likely therefore that there was significant mortality even among those who could not be traced. This excess mortality should be accounted for in calculating mortality rates [20,21]. The likely impact of LTFU to our estimate for mortality is therefore an underestimation of the true value but it is unlikely to affect the predictors except were ascertainment of outcome differed amongst the subgroups (when LTFU is itself the real factor responsible for mortality).

The major reason for defaulting was lack of personal motivation. After our conversation with them, they all promised to return to care. Extending this study for a couple of months would investigate if they did indeed return. Patient education on the necessity for a lifelong and regular care is therefore crucial. Given that the clinic has a limited human resource, a mass or group education of these patients will appear to be the cost-effective means of going around this obstacle. Four of them were receiving alternative remedies from traditional practitioners. Traditional medicine is a common but a disorganised practice in the country. It is likely that many more are using alternative therapy even those in care but data on this practice was not routinely collected by health workers. Over 80% of Africans do rely on traditional medicine or indigenous knowledge to meet their health needs for one or another reason [28]. There were eleven of such tradipractioners receiving care in this cohort. Financial problems were less of an issue given that many aspects of care have become free of charge compared to the era before 2007 [14].

This study had a couple of draw-backs that may impact on its quality. Its retrospective design made it liable to incompleteness of information collected. The mortality and LTFU rates calculated are an underestimation of the expected. Our data are hospital-based thus making it somehow difficult to tell if our results can be extrapolated to the general population. The study however can be credited for its relatively sufficient sample size and its cohort design that allows calculating incidence of mortality and LTFU.

## Conclusion

Access to HIV care has been scaled up in recent years but the outcome of patients in terms of mortality and loss to follow up our clinic is only beginning to improve since 2010. An extension of the study for a couple of years is necessary to confirm the down trend in Mortality and LTFU. Men, late presentation, anaemia and drug regimen were the main factors strongly associated with mortality while the number of patients enrolled and how they were monitored were the major determinants of LTFU. While patients need to be motivated to present at an early stage and continue a lifelong follow up, interventions to address factors associated with high mortality and LTFU should be implemented for optimal results in patient care.

## Competing interests

I declare that I have no competing interests.

## Authors’ contributions

CEB: The project conception, literature search, data abstraction and translation from French into English, data entry and analyses and the write-up. JW: The project conception, extensive comments, approval for publication. (She was my personal tutor and project supervisor as an MSc student at LSHTM in 2012). MB, GES and BK: Extensive revision and critical appraisal of manuscript as well as local supervision of project. All authors read and approved the final manuscript.

## Authors’ information

CEB is a holder of an MD degree from The University of Yaounde 1 and of an MSc degree in Control of Infectious Diseases from LSHTM. Currently, he is Chief Medical Officer of the Bare Sub-divisional Medical Centre and a visiting physician at the HIV Clinic of the Nkongsamba Regional Hospital in, Cameroon.

JW is a senior lecturer at LSHTM in the Faculty of Infectious and Tropical Diseases where she is currently the research coordinator at the Department of Disease Control.

MB is a researcher at the Department of Global Health of The University of Washington, USA.

GES is a public health physician who currently serves as the National Programme Officer for Immunization in the WHO country office, Cameron.

BK is the Head of Department of Public Health at The University of Douala, Cameroon. He is also the current board chairman of the Regional Hospital of Nkongsamba, Cameroon.
